# Increased Risk of Developing Digestive Tract Cancer in Subjects Carrying the *PLCE1* rs2274223 A>G Polymorphism: Evidence from a Meta-Analysis

**DOI:** 10.1371/journal.pone.0076425

**Published:** 2013-10-07

**Authors:** Xunlei Zhang, Yangmei Zhang, Dongying Gu, Chunxiang Cao, Qi Zhang, Zhi Xu, Yongling Gong, Jinfei Chen, Cuiju Tang

**Affiliations:** 1 Department of Oncology, Nanjing First Hospital, Nanjing Medical University, Nanjing, China; 2 Department of Oncology, Xuzhou Hospital Affiliated to Medical College of Southeast University and Xuzhou Central Hospital, Xuzhou, China; Shanghai Jiao Tong University School of Medicine, China

## Abstract

**Background:**

To date, the association between phospholipase C epsilon 1 (*PLCE1*) rs2274223 A>G and risk of digestive tract cancer (DTC) remains inconclusive. To derive a more precise estimation of the association, we conducted a meta-analysis on all eligible case–control studies involving 8281 cases and 10,532 controls.

**Methods:**

A comprehensive search was conducted to identify all eligible studies of *PLCE1* rs2274223 polymorphism and digestive tract cancer risk. The pooled odds ratio (OR) and the 95% confidence interval (95% CI) were calculated using a fixed or random effect model. Heterogeneity, publication bias, and sensitivity analysis were also explored.

**Results:**

Overall, the *PLCE1* rs2274223 A>G polymorphism was associated with risk of DTC in all genetic models (GA vs. AA: OR = 1.21, 95% CI = 1.14–1.29, *P*<0.001; GG vs. AA: OR = 1.30, 95% CI = 1.06–1.60, *P* = 0.012; GG/GA vs. AA: OR = 1.20, 95% CI = 1.10–1.32, *P*<0.001; GG vs. GA/AA: OR = 1.21, 95% CI = 1.01–1.46, *P* = 0.040). The recessive model did not reach statistically significance when the *P* values were Bonferroni corrected to 0.0125. In the stratified analysis by cancer type, ethnicity, and source of controls, significantly increased risk was observed for esophagus cancer, Asians in three genetic models (heterozygote comparison, homozygote comparison and dominant model), population-based studies in all genetic models, and for gastric cancer in the heterozygote comparison and dominant model after Bonferroni correction. However, in the subsite of gastric cancer, no significant association was found either in cardia or non-cardia gastric cancer.

**Conclusion:**

Our study indicated that *PLCE1* rs2274223 A>G polymorphism was significantly associated with increased risk of DTC, especially among Asian populations. Due to some minor limitations, our findings should be confirmed in further studies.

## Introduction

Digestive tract cancer (DTC) referring to a group of malignancies (e.g., located in oral cavity, pharynx and larynx, esophagus, stomach, small and large intestines) is the most common cancer worldwide. It has been estimated that there were nearly 316,970 new diagnosed cases and 147,150 deaths caused by DTC in the United States in 2011 [1]. Esophageal, gastric and colorectal cancers are the leading causes of cancer-related death in Eastern Asian countries [2], [3]. Therefore, identification of potential risk factors for DTC may contribute to the prevention and early diagnoses of these lethal cancers.

It has been suggested that DTC carcinogenesis is a combined effect of multiple factors, which contains environmental factors, dietary habits and inherited susceptibility [4], [5]. Alcohol consumption and tobacco smoke are the well-recognized risk factors for DTC [6]. Despite the high prevalence of *Helicobacter pylori* (*HP*) infection in gastric cancer, human papillomavirus (*HPV*) is recognized as a major risk factor for oropharygeal cancer [7], [8]. Recently, accumulative evidence has shown that genetic factors, especially gene polymorphisms, which involve in multiple biological pathways, such as carcinogen metabolism, apoptosis, DNA repair, cell cycle regulation, and other cellular processes, play important roles in the etiology of DTC [9], [10], [11], [12].

The *PLCE1* gene, located on chromosome 10q23, is a unique member of the phospholipase family [13]. *PLCE1* encodes the phospholipase C epsilon 1 (*PLCε1*) that catalyses the hydrolysis of phosphatidylinositol-4,5-bisphosphate into the secondary messengers inositol 1,4,5-trisphosphate and diacylglycerol (DAG), which participate in cell growth, differentiation and gene expression [14]. *PLCE1* has been speculated to be a effector of small GTPases of the Ras, Rap and Rho families, and contains a guanine nucleotide exchange factor domain for Ras-like small GTPases at its N-terminus and two Ras-binding domains at its C-terminus [13], [14], [15], [16]. Recent studies have reported that *PLCE1* plays crucial roles in carcinogenesis and progression of several types of cancers, including cancers of the intestine, skin, bladder, colorectal and head and neck [17], [18], [19], [20], [21].

Rs2274223 (A>G) is a non-synonymous single nucleotide polymorphism (SNP) located in 26th exon of the *PLCE1* gene and result in the amino acid change from histidine (His) to arginine (Arg) at codon 1927 of *PLCE1*. In 2010, two large-scale genome-wide association studies (GWASs) simultaneously reported that the new and notable low-penetrance susceptibility locus rs2274223 was strongly associated with risk of esophageal squamous cell carcinoma (ESCC) and gastric cardia adenocarcinoma (GCA) in Chinese population [22], [23]. Recent studies indicated that rs2274223 (A>G) was associated with an increased risk of most DTC, such as cancers of oral cavity, pharynx and larynx, esophagus, and stomach [24], [25], [26], [27], [28], [29], [30], [31]. Interestingly, another study proved that rs2274223 was associated with a protective effect against colorectal cancer (CRC) in a Chinese population [32]. Otherwise, as reported in a Dutch population and a South African population, it was unlikely that the *PLCE1* rs2274223 SNP plays a role in esophageal adenocarcinoma (EAC) or ESCC susceptibility [33], [34]. Besides, there was a study focused on the associations between the rs2274223-G allele and the prognosis of gastric cancer patients, suggesting that individuals carrying rs2274223 AG/GG genotypes had a higher survival rate than those carrying the AA genotype [35].

To date, the association between the rs2274223 (A>G) and the susceptibility of DTC are inconclusive in different cancer types and ethnicities, partially because of the different effects of the polymorphism on variants of DTC risk and the relatively small sample size in each of published studies. Hence, we conducted a meta-analysis on all eligible case–control studies involving 8281 cases and 10,532 controls to estimate the overall DTC risk of the rs2274223 (A>G) polymorphism.

## Materials and Methods

### Identification and Eligibility of Relevant Studies

We searched PubMed and Embase (updated to 28th February 2013) using the following search terms: “rs2274223”, “*PLCE1*”, “10q23”, “genetic susceptibility”, “SNP”, “polymorphism” or “variation”, and “cancer” or “carcinoma” or “neoplasia”. The search was limited to English-language articles. We also used a hand search of references of original studies on this topic in order to identify additional studies. Studies included in our meta-analysis have to meet the following inclusion criteria: (a) evaluated the *PLCE1* rs2274223 polymorphism and cancer risk, (b) used a case–control design and (c) contained available genotype frequency.

### Data Extraction

Two investigators independently extracted data and reached a consensus on all the items in cases of discordance. For each eligible study, the following data were extracted: the first author’s name, year of publication, ethnicity, country of origin, cancer type, value of Hardy–Weinberg equilibrium (HWE), source of controls, genotyping method,and numbers of genotyped cases and controls. Ethnic descents were categorized as European and Asian (the only one African study was excluded). For studies including subjects of different sites of gastric cancer (cardia and non-cardia), data were extracted separately whenever possible.

### Statistical Analysis

The strength of the association between the *PLCE1* rs2274223 polymorphism and cancer risk was assessed by the odds ratio (OR) and the 95% confidence interval (95% CI). Pooled ORs were obtained from the combination of individual studies by heterozygote comparison (GA vs. AA), homozygote comparison (GG vs. AA), a dominant model (AA/GA vs. GG), and a recessive model (AA vs. GA/GG). The significance of pooled ORs was determined using the Z-test. Bonferroni correction was utilized for multiple testing. Because multiple comparisons were performed 4 times respectively, the *P* value lesser than 0.05/4 (0.0125) was accepted for statistical significance after Bonferroni correction. Both the Cochran’s Q statistic to test for heterogeneity and the I2 statistic to quantify the proportion of the total variation due to heterogeneity were calculated to estimate heterogeneity among the included studies [36], [37]. If the *P* value of the Q test was <0.05, indicating a lack of heterogeneity across studies, the summary OR estimate of each study was calculated by the fixed effects model (the Mantel–Haenszel method) [38]. Otherwise, random effects model (the DerSimonian and Laird method) was used [39]. Stratified analyses were also performed by cancer type, ethnicity, source of controls and site of gastric cancer. Sensitivity analyses were performed to evaluate the stability of the results by deleting a single study in the meta-analysis each time to show the influence of the individual data set to the pooled OR. Funnel plots and Egger’s linear regression test were used to assess the potential publication bias [40]. All analyses were performed using Stata software (version 8.2; StataCorp LP, College Station, TX), using two-sided *P* values.

## Results

### Characteristics of Studies

**Figure 1:**
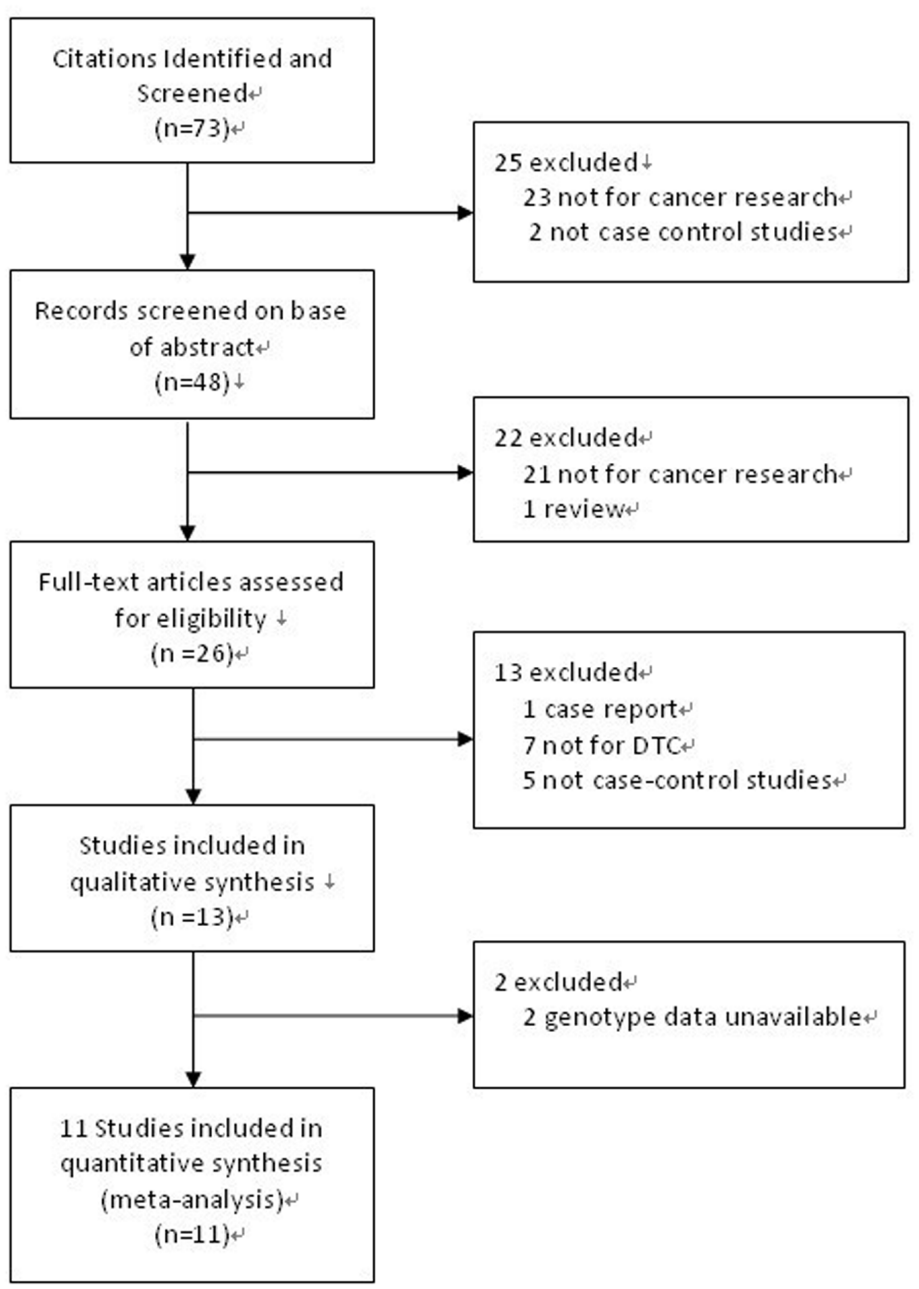
Flow diagram summarizing the search strategy.

Figure 1 illustrates the study selection process. A total of eleven eligible studies involving 8281 cases and 10,532 controls met all inclusion criteria and were included in the pooled analyses [24], [25], [26], [27], [28], [29], [30], [31], [32]. The main characteristics of these studies are shown in Table 1. The distribution of genotypes in the controls was consistent with the Hardy–Weinberg equilibrium for all selected studies. All studies were case–control studies, including one gastric cancer and esophageal cancer study, two gastric cancer studies, five esophageal cancer studies, one colorectal cancer study and two head and neck (oral cavity, pharynx and larynx) cancer studies. Among these gastric cancer studies, two studies provided genotype information of cardia and non-cardia gastric cancer. There were seven studies involved Asian descendants, three studies involved European descendants and one study involved African descendants. Controls in seven studies were population-based and other four studies were hospital-based. The TaqMan assay was performed in seven of the eleven studies.

**Table 1:** Characteristics of literatures included in the meta-analysis.

Author	year	country	Ethnicity	Source ofcontrols	Cancertype	Genotypingmethod	cases			controls			HWE
							AA	AG	GG	AA	AG	GG	
Ma	2011	America	European	Hospital	HNC	TaqMan	477	506	114	504	474	111	0.980
Yuan	2012	China	Asian	Population	HNC	TaqMan	301	170	30	547	300	32	0.243
Li	2012	China	Asian	Hospital	CRC	MassARRAY	155	71	5	180	92	20	0.089
Zhang	2011	China	Asian	Population	GC	TaqMan	867	664	134	1122	643	83	0.451
					Cardia		373	355	84				
					Non-cardia		421	259	40				
Palmer	2012	America	European	Population	GC	TaqMan	239	288	68	240	273	73	0.187
					Cardia		115	118	25				
					Non-cardia		124	170	43				
					EC	TaqMan	74	68	17	86	107	17	0.187
Zhou	2012	China	Asian	Population	EC	PCR-LDR	248	227	42	291	191	28	0.646
Wang	2012	China	Asian	Population	GC	TaqMan	600	399	60	791	390	59	0.224
Hu	2012	China	Asian	Population	EC	TaqMan	594	400	67	754	399	58	0.577
DuraGuBye	201220122012	NetherlandsChinaSouth African	EuropeanAsianAfrican	PopulationHospitalHospital	ECECEC	RT-PCRMassARRAYTaqMan	160202218	154147338	3030116	279233612	247119819	5419276	0.9500.4570.943

**Abbreviations:** HWE, Hardy–Weinberg equilibrium; HNC, head and neck (oral cavity, pharynx and larynx) cancer; EC, esophageal cancer; GC, gastric cancer; CRC, colorectal cancer.

### Quantitative Synthesis

The evaluation of the association between the *PLCE1* rs2274223 polymorphism and the susceptibility to digestive tract cancer is presented in Table 2. Overall, the variant G allele of rs2274223 A>G could significantly increase the risk of cancer in all genetic models (heterozygote comparison, GA vs. AA: OR = 1.21, 95% CI = 1.14–1.29, *P*<0.001, I^2^ = 35.70%; homozygote comparison, GG vs. AA: OR = 1.30, 95% CI = 1.06–1.60, *P* = 0.012, I^2^ = 65.60%; dominant model, GG/GA vs. AA: OR = 1.20, 95% CI = 1.10–1.32, *P*<0.001, I^2^ = 57.60%; recessive model, GG vs. GA/AA: OR = 1.21, 95% CI = 1.01–1.46, *P* = 0.040, I^2^ = 60.00%). However, after Bonferroni correction, this association in recessive model did not reach statistically significance when the *P* values were correction to 0.0125.

**Table 2:** Stratified analyses of the *PLCE1* rs2274223 A>G polymorphism on cancer risk.

Variables	N^b^	case/control	GA vs. AA				GG vs. AA				GG/GA vs. AA				GG vs. GA/AA			
			OR	*P*(Z)	*P*(Q)	I^2^	OR	*P*(Z)	*P*(Q)	I^2^	OR	*P*(Z)	*P*(Q)	I^2^	OR	*P*(Z)	*P*(Q)	I^2^
Total	11	8281/10532	1.21(1.14–1.29)	<0.001	0.113	35.70%	1.30(1.06–1.60)^c^	0.012	0.001	65.60%	1.20(1.10–1.32)^c^	<0.001	0.009	57.60%	1.21(1.01–1.46)^c^	0.040	0.005	60.00%
GC	3	3319/3674	1.29(1.17–1.42)	<0.001	0.223	33.30%	1.40(0.87–2.25)^c^	0.171	0.003	82.60%	1.32(1.20–1.46)	<0.001	0.063	63.80%	1.28(0.83–1.98)^c^	0.267	0.006	80.50%
EC	6	3133/4598	1.22(1.10–1.34)	<0.001	0.142	39.6%	1.31(1.10–1.55)	0.002	0.419	0%	1.24(1.13–1.36)	<0.001	0.104	45.20%	1.20(1.02–1.40)	0.027	0.535	0%
Others^a^	3	1829/2260	1.07(0.93–1.22)	0.351	0.525	0%	0.94(0.48–1.87)^c^	0.867	0.008	79.10%	1.07(0.94–1.21)	0.330	0.217	34.60%	0.93(0.48–1.81)^c^	0.826	0.009	78.80%
Ethnicity																		
Asian	7	5414/6360	1.28(1.18–1.38)	<0.001	0.214	28.00%	1.51(1.13–2.00)^c^	0.005	0.015	62.20%	1.29(1.15–1.44)^c^	<0.001	0.046	53.20%	1.39(1.06–1.80)^c^	0.016	0.027	57.80%
European	3	2195/2465	1.07(0.95–1.21)	0.296	0.583	0%	1.03(0.84–1.26)	0.787	0.879	0%	1.06(0.94–1.19)	0.324	0.598	0%	1.00(0.83–1.21)	0.976	0.945	0%
Control source																		
Population	7	5901/7064	1.23(1.14–1.33)	<0.001	0.088	45.50%	1.43(1.13–1.81)^c^	0.003	0.020	60.30%	1.24(1.11–1.39)^c^	<0.001	0.029	57.40%	1.34(1.17–1.55)	<0.001	0.056	51.20%
Hospital	4	2380/3468	1.15(1.03–1.29)	0.017	0.307	16.90%	1.08(0.73–1.59)^c^	0.717	0.022	68.90%	1.14(1.02–1.28)	0.017	0.067	58.10%	1.02(0.73–1.42)^c^	0.931	0.045	62.80%
Subsite of GC																		
Cardia	2	1070/2434	1.24(0.68–2.26)^c^	0.479	0.001	91.20%	1.50(0.36–6.13)^c^	0.580	<0.001	95.60%	1.27(0.61–2.63)^c^	0.528	<0.001	94.60%	1.38(0.43–4.40)^c^	0.586	<0.001	93.90%
Non–cardia	2	1057/2434	1.11(0.95–1.30)	0.186	0.507	0%	1.22(0.91–1.63)	0.189	0.690	0%	1.12(0.97–1.30)	0.121	0.623	0%	1.14(0.86–1.50)	0.371	0.491	0%

a Contains two HNC and one CRC studies.

b Number of comparisons. One of these studies consisted of GC and EC.

c Random-effects model was used when *P* value for heterogeneity test <0.05; otherwise, fixed-effects model was used.

*P* (Q): *P*-value of Q-test for heterogeneity test.

*P* (Z): *P*-value of Z-test for overall OR. *P*<0.0125 defined as the significance threshold after Bonferroni correction.

**Figure 2:**
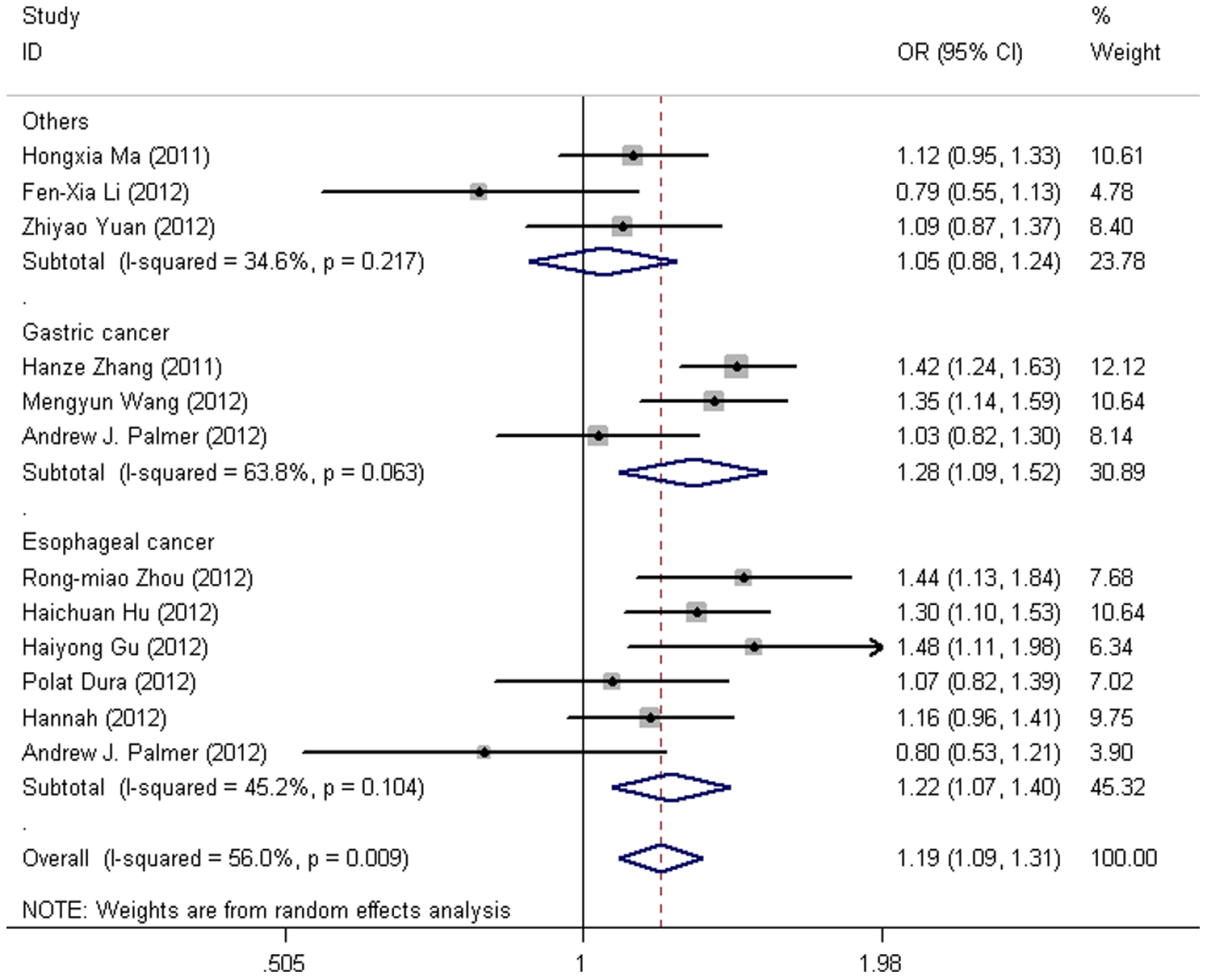
Forest plot from the meta-analysis of *PLCE1* rs2274223 A>G polymorphism and the risk of DTC stratified by cancer types using dominant genetic model.

Additionally, in the analysis stratified by cancer types (Figure 2), the *PLCE1* rs2274223 polymorphism was significantly linked to a higher risk for esophageal cancer (GA vs. AA: OR = 1.22, 95% CI = 1.10–1.34, *P*<0.001, I^2^ = 39.60%; GG vs. AA: OR = 1.31, 95% CI = 1.10–1.55, *P* = 0.002, I^2^ = 0%; GG/GA vs. AA: OR = 1.24, 95% CI = 1.13–1.36 *P*<0.001, I^2^ = 45.20%; GG vs. GA/AA: OR = 1.20, 95% CI = 1.02–1.40, *P* = 0.027, I^2^ = 0%). However, the recessive model did not reach statistically significance when the *P* values were Bonferroni corrected. We also observed increased susceptibility of gastric cancer in heterozygote comparison (GA vs. AA: OR = 1.29, 95% CI = 1.17–1.42, *P*<0.001, I^2^ = 33.30%) and dominant model (GG/GA vs. AA: OR = 1.32, 95% CI = 1.20–1.46, *P*<0.001, I^2^ = 63.80%). No significant associations were found in the “others” group (colorectal cancer and head and neck cancer).

**Figure 3:**
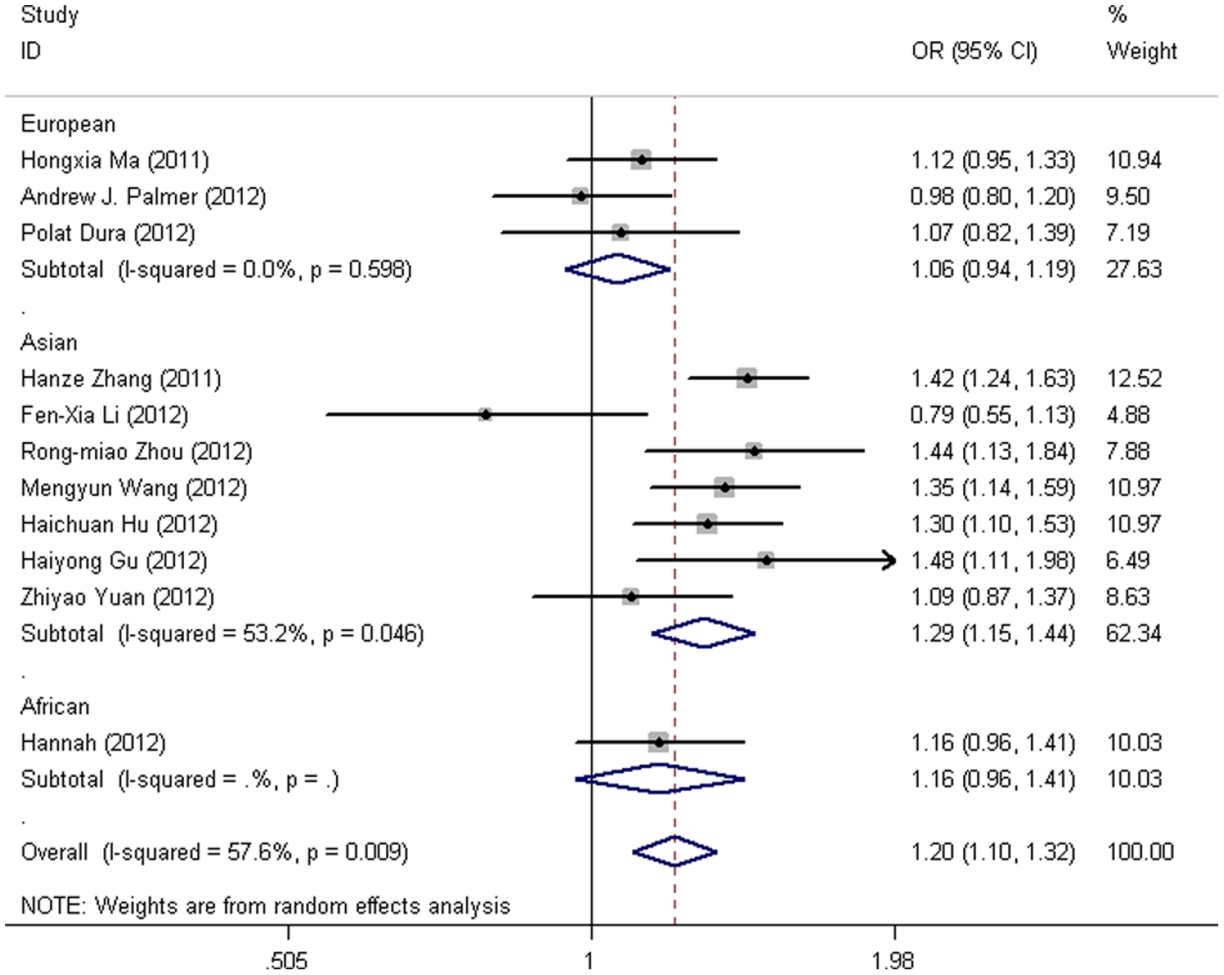
Forest plot from the meta-analysis of *PLCE1* rs2274223 A>G polymorphism and the risk of DTC stratified by ethnicity using dominant genetic model.

For the Asian group, every genetic comparison produced significantly increased risks (GA vs. AA: OR = 1.28, 95% CI = 1.18–1.38, *P*<0.001, I^2^ = 28.00%; GG vs. AA: OR = 1.51, 95% CI = 1.13–2.00, *P* = 0.005, I^2^ = 62.20%; GG/GA vs. AA: OR = 1.29, 95% CI = 1.15–1.44, *P*<0.001, I^2^ = 53.20%; GG vs. GA/AA: OR = 1.39, 95% CI = 1.06–1.80, *P* = 0.016, I^2^ = 57.80%), but the recessive model did not reach statistically significance Bonferroni correction, whereas no significant associations were detected among the European group (Figure 3). Considering the control source, studies with population-based controls showed elevated risks in four genetic comparisons (heterozygote comparison, GA vs. AA: OR = 1.23, 95% CI = 1.14–1.33, *P*<0.001, I^2^ = 45.50%; homozygote comparison, GG vs. AA: OR = 1.43, 95% CI = 1.13–1.81, *P* = 0.003, I^2^ = 60.30%; dominant model, GG/GA vs. AA: OR = 1.24, 95% CI = 1.11–1.39, *P*<0.001, I^2^ = 57.40%; recessive model, GG vs. GA/AA: OR = 1.34, 95% CI = 1.17–1.55, *P*<0.001, I^2^ = 51.20%). By contrast, studies with hospital-based controls only presented significant associations in heterozygote comparison (GA vs. AA: OR = 1.15, 95% CI = 1.03–1.29, *P* = 0.017, I^2^ = 16.90%) and dominant model comparisons (GG/GA vs. AA: OR = 1.14, 95% CI = 1.02–1.28, *P* = 0.017, I^2^ = 58.10%), but this did not reach statistically significance when the *P* value was Bonferroni corrected. In the subgroup analysis by tumor sites of gastric cancer, there was no significant association detected in any genetic comparisons either in cardia or non-cardia gastric cancer.

### Test of Heterogeneity

When evaluating the association between the *PLCE1* rs2274223 polymorphism and the susceptibility to DTC, we found that there was significant heterogeneity for the homozygote comparison (GG vs. AA: *P*_heterogeneity_ = 0.001, I^2^ = 65.60%), dominant model comparison (GG/GA vs. AA: *P*_heterogeneity_ = 0.009, I^2^ = 57.60%) and recessive model comparison (GG vs. GA/AA: *P*_heterogeneity_ = 0.005, I^2^ = 60.00%) but not for the heterozygote comparison (GA vs. AA: *P*_heterogeneity_ = 0.113, I^2^ = 35.70%). Thus, we assessed the source of heterogeneity for the dominant model comparison by examining cancer type, ethnicity, source of controls and genotyping method. Meta-regression analyses showed that none of these concomitant variables could account for the substantial heterogeneity observed (ethnicity: *P* = 0.215, genotyping method: *P* = 0.925, cancer site: *P* = 0.286 and source of controls: *P* = 0.408).

### Sensitivity Analysis

In order to reflect the influence of the individual dataset to the pooled ORs, we deleted a single study involved in the meta-analysis each time, but the corresponding pooled ORs were not altered materially (data not shown), suggesting that our results were statistically robust.

### Publication Bias

**Figure 4:**
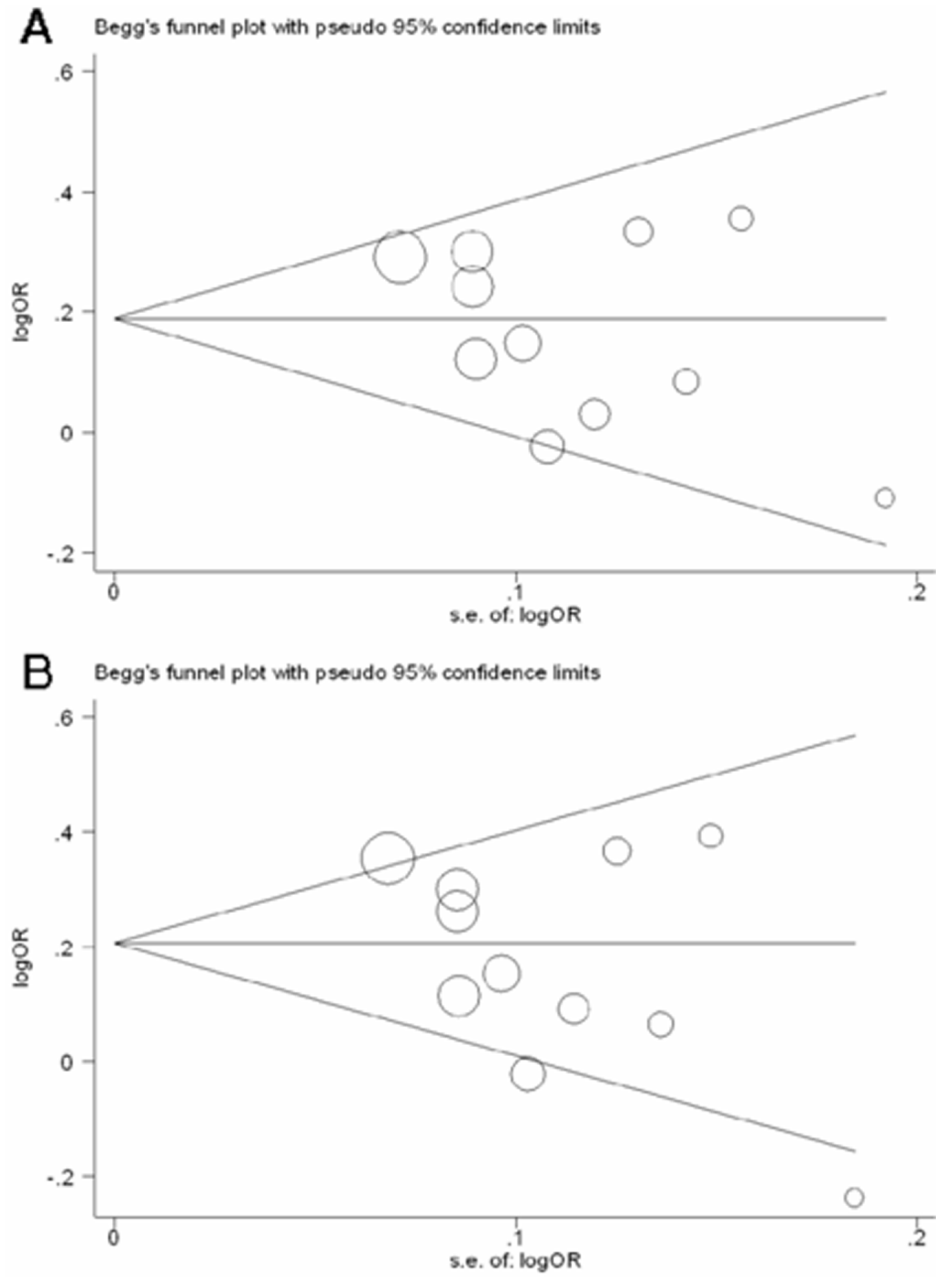
Begg’s funnel plot of publication bias test. (A) GA vs. AA; (B) GG/GA vs. AA. Each point represents a separate study for the indicated association. Log (OR), natural logarithm of OR. Horizontal line, mean effect size.

Begg’s funnel plot and Egger’s test were performed to assess the publication bias of the literature. The shape of the funnel plots did not reveal any evidence of obvious asymmetry (Figure 4 shows the funnel plot of the overall GA vs. AA and GG/GA vs. AA comparisons). Then, the Egger’s test was used to provide statistical evidence of funnel plot symmetry. Results still did not show any obvious evidence of publication bias (GA vs. AA: *P* = 0.205; GG/GA vs. AA: *P* = 0.137).

## Discussion

In the current meta-analysis, we ascertained that the *PLCE1* rs2274223 A>G polymorphism was significantly associated with increased DTC risk, especially with gastric cancer and esophageal cancer. To our knowledge, this is the first study to investigate the association between the *PLCE1* rs2274223 A>G polymorphism and the risk of DTC across different ancestries.

The PLCE1 protein is a member of the phospholipase family and functions as an effector of guanosine triphosphatases (GTPases) such as Ras, Rap1 and Rap2 involving regulation of cell growth, differentiation and apoptosis [14], [21]. *PLCE1* also interacts with IQ-domain GTPase-activating protein 1 (IQGAP1) [41], which plays an important role in angiogenesis and is expressed in the endothelial cells, where it binds VEGFR2, an important factor for the endothelial cell rearrangement and migration [42]. This polymorphism rs2274223 causes an amino acid change from histidine to arginine in the *PLCE1* protein calcium-dependent lipid-binding (C2) domain.

Wang et al. and Abnet et al. simultaneously reported the strong association of the new and notable low-penetrance susceptibility locus rs2274223 with the increased risk of ESCC and GCA in the Chinese population by two large-scale GWASs [22], [23]. However, it was unlikely that the *PLCE1* rs2274223 SNP played a role in EAC or ESCC susceptibility in American and Dutch [26], [33]. Further observation showed that the rs2274223-G allele had a stronger effect on female and GCA than male and non-cardia GC [25]. Luo et al. found that individuals carrying *PLCE1* rs2274223 AG/GG genotypes had a higher survival rate than those carrying the AA genotype, which suggested that the rs2274223-G allele may be associated with the prognosis of the gastric cancer patients [35]. Yuan et al. and Ma et al. also performed GAWSs and confirmed that rs2274223 was associated with a significantly increased risk of head and neck cancer [24], [31]. Furthermore, Li et al. proved that rs2274223 was associated with a decrease risk of CRC in a Chinese population [32].

Although many epidemiological studies regarding the *PLCE1* rs2274223 polymorphism on the risk of variants of DTC had been conducted, the results were conflicting and inconclusive because of various reasons, such as different ethnicities, resident areas, sample size, environmental factors and diet habits. To provide a more comprehensive analysis on the association, we carried out this meta-analysis based on eleven case–control studies with 18,813 participants and indicated that G allele of the *PLCE1* rs2274223 A>G polymorphism was associated with increased risk of DTC.

In the analysis stratified by cancer type, we observed increased susceptibility to esophageal cancer in the three genetic comparisons (heterozygote comparison, homozygote comparison and dominant model) and to gastric cancer in the heterozygote comparison (GA vs. AA) and the dominant model comparison (GG/GA vs. AA) after Bonferroni correction. The results were consistent with the conclusions of most previous studies [22], [23], [25], [26], [27], [28], [29], [30], except for three studies about esophageal cancer in American, Dutch and South African [26], [33], [34], partially because of the different ethnicities and the relatively small sample size in the three studies.

When compared by ethnicity, statistically significantly increased risks were found among Asians for three genetic comparison (heterozygote comparison, homozygote comparison and dominant model) but not Europeans after Bonferroni correction. Although the exact mechanism for these ethnic differences is still unknown, one possible reason is due to differences in genetic backgrounds and in the environmental and lifestyle context (such as dietary habits, alcohol consumption and tobacco smoke) [43]. In addition, because of the gene-gene interaction, the influence of the *PLCE1* rs2274223 A>G polymorphism might be masked or magnified by the presence of other genes which were unidentified yet in the development of cancer. Other factors such as selection bias, different matching criteria and limited number of studies with available data may have insufficient statistical power to detect a slight difference and may also generate a fluctuated risk estimate.

We also observed a significantly increased DTC risk among studies using population-based controls in every genetic model, but not using hospital-based controls. Some biases may exist in hospital-based studies, for such controls may represent a sample of an ill-defined reference population instead of the general population, particularly when the genotypes investigated were associated with the disease that the hospital-based controls may have. Thus, a proper and representative cancer-free control subject is very important in reducing biases in such genotype association studies.

Several previous studies showed that the association between rs2274223 and the risk of gastric cancer was stronger in the cardia than in the non-cardia gastric cancer [22], [23], [25], [28]. Our study also explored the association with gastric cancer differed by anatomic subsite. Unfortunately, no significant association was detected in any genetic comparisons either in cardia or non-cardia gastric cancer. The null results might be due to the limited number of studies with available data on these characteristics and the cases of the eligible studies were from the different ethnicities, which had insufficient statistical power to detect a slight effect.

Some limitations of this meta-analysis may have affected the objectivity of the conclusions and should be considered in interpreting the results. First, the quantity of published studies was not sufficiently large for a comprehensive analysis, and the lack of original data in some studies limited our further evaluation of potential interactions, such as ethnicity, anatomic subsite and pathological subtype of cancer. Second, although perfect searching strategy was used to identify eligible studies for current meta-analysis, it was still possible that a few studies meeting inclusion criteria were not included. Third, the overall outcomes were based on unadjusted estimates, while a more precise evaluation should be conducted if more detailed individual data were available, such as age, sex, histological types and *HP* infection. Lacking of information may cause serious confounding bias.

In spite of these limitations, our present meta-analysis also had some advantages. First, we estimated the association conclusively between the *PLCE1* rs2274223 A>G polymorphism and DTC risk, and further showed the significant association especially among Asians rather than Europeans. This study may also provide a potential genetic marker and a new insight into the etiology of DTC. Second, we pooled a substantial number of cases and controls from different studies, which greatly increased the statistical power of the analysis. Third, no publication biases were detected, which indicated that the results were likely unbiased.

In conclusion, our meta-analysis suggests that the *PLCE1* rs2274223 A>G polymorphism is associated with DTC risk, especially with gastric cancer and esophageal cancer. The *PLCE1* rs2274223 A>G polymorphism is an independent risk factor for the development of DTC, and will probably be a potential therapeutic target for new drugs. Nevertheless, larger and well-designed multicentric studies including other potential DTC risks (such as gene-gene, gene-environmental interactions) should be carried out to validate our findings. Furthermore, research based on the non-DTCs (such as hepatic cellular cancer, lung cancer, breast cancer etc.) should be performed to explore the association between *PLCE1* gene polymorphisms and cancer risks.

## Supporting Information

Checklist S1 A PRISMA checklist for this meta-analysis.
(DOC)
